# Vaccine Hesitancy, Anti-Vax, COVID-Conspirationism: From Subcultural Convergence to Public Health and Bioethical Problems

**DOI:** 10.3389/fpubh.2022.877490

**Published:** 2022-05-09

**Authors:** Andrea Raballo, Michele Poletti, Antonio Preti

**Affiliations:** ^1^Section of Psychiatry, Clinical Psychology and Rehabilitation, Department of Medicine, University of Perugia, Perugia, Italy; ^2^Center for Translational, Phenomenological and Developmental Psychopathology (CTPDP), Perugia University Hospital, Perugia, Italy; ^3^Department of Mental Health and Pathological Addiction, Child and Adolescent Neuropsychiatry Service, Azienda USL-IRCCS di Reggio Emilia, Reggio Emilia, Italy; ^4^Department of Neuroscience, University of Turin, Turin, Italy

**Keywords:** COVID-19 denial, informed consent, compulsory treatment, anti-vaccine campaign, conspirationism, vaccine hesitancy

## Introduction

Vaccine hesitancy is not in itself a novel social and individual phenomenon, yet the ongoing COVID-19 pandemic is associated to increasing degrees of widespread sociopolitical weaponization of such attitude, becoming a major threat to the progress and success of vaccination campaigns (1–3).

Behavioral vaccine-hesitancy might be due to heterogeneous motivations. The majority of people simply adhere to an over-cautious “wait-and-see” attitude, due to presumed, possible unforeseeable long-term effects of fast-authorized novel vaccines; a minority of people adhere to an anti-vaccine activism (usually labeled as Anti-Vax), which proactively opposes vaccinations denying the existence of COVID-19 or ascribing bizarre, deliberately malignant biopsychosocial effects to current vaccines (4–6) and boosting trust in fake and irrational beliefs ^1^.

## Public Health Consequences at Group- and Individual Levels

On a public health perspective, the most extreme, impermeable side of the Anti-Vax spectrum is posing a plateau to the vaccination rate and allegedly retarding the reach of a possible herd immunity (7). At the same time, Anti-Vax activists are often publicly blamed as infectors being the major cause of infective surges, thereby becoming the new, transnational political scapegoat for cumulative public health inefficiencies and related socio-economic shock-waves.

Moreover, at the individual level, there is increasing reporting of another phenomenon that warrants further reflection: although not yet quantified by focused surveys, there is reporting of hospitalized unvaccinated COVID-19-deniers that refuse the best therapies and even intensive care treatment if needed^2^^,^^3^. This phenomenon has recently led the Italian Society of Anesthesiology, Analgesia, Resuscitation and Intensive Care to officially discuss the ethical issues raised by this hazardous self-threatening behavior, given that, in some cases, this has led to the death of the hospitalized patient^4^, with additional psychological burden in already overwhelmed healthcare workers.

This potentially lethal, self-threatening behavior is apparently expressed without manifest signs of suicidal intention or documented psychopathology. Such para-suicidal behavior in COVID-19 deniers evokes some features of faith-based (e.g., Peoples Temple in Guyana, Order of the Solar Temple in Switzerland, France and Canada, Heaven's Gate in Santa Fe, USA) (8) and ideologically-based suicides (e.g., suicidal terrorism) (9), since it is enacted on the background of shared, specific worldviews. However, while these latter suicidal behaviors are explicitly based on an envisioned, post-mortem scenario of eternal glory, salvation or political revolution, the acceptance of a serious, life-endangering risk due to the refusal of suitable therapies for an illness whose existence is denied is far less comprehensible. Indeed, shifting from a proclivity to entertain conspiracy beliefs to the point of refusing appropriate therapy is a significant psycho-behavioral step. Concretely, it means to explicitly enact a life-threatening behavior on the ideological basis of Anti-Vax/COVID-denialist narrative.

One plausible explanation is that these patients really mistrust the existence of COVID-19 and therefore do not realize that they could die if not adequately treated. This hypothesis presupposes a weakening of the reality testing and an ongoing para-delusional thinking which is not amenable to change in light of massive conflicting evidence as own's physical symptoms requiring hospitalizations, treatment indications of the medical staff, presence of other hospitalized patients with similar health conditions, ongoing societal measures to contain the pandemic; indeed, if these features could be neglected through the echo-chamber phenomenon (10) while healthy and at home, they are more difficult to ignore when ill and hospitalized.

This agrees with the alleged importance of maladaptive personality features (such as schizotypal odd beliefs) and poorer reality testing in determining a higher proneness to entertain conspiracy beliefs (11–13). This suggests that individual psychotic-like features (e.g., odd beliefs, poor reality testing, biased thinking not amenable to change in light of conflicting or disconfirmatory evidence) are likely to contribute to the enactment of COVID-related conspiracy beliefs to their utmost consequences (including self-threatening therapeutic refusal).

## Bioethical Dilemma: Illness Denial and Informed Consent

Overall, the contiguity of a fixed belief which is incorrigible despite massive, surrounding disconfirmatory evidence with a psychotic-like mental state, is particularly critical, given that even the Diagnostic and Statistical Manual of Mental Disorders 5th Edition (DSM-5) as well as the International Classifications of Diseases 11th Revision (ICD-11) emphasize the distinction between delusional and culturally-grounded beliefs, assuming that delusions generally involve beliefs not ordinarily accepted by other members of the person's culture or subculture. Yet, the high prevalence of some type of COVID-denialism among a significant worldwide minority of the population (14) makes it *de facto* a culturally-grounded belief. Nonetheless, if people who deny the existence of COVID-19 decline urgent, non-deferrable lifesaving interventions because they are in a delusional-like mental state (i.e., a psychotic state of mind), compulsory treatment might be legitimately applied because the individual's ability to make decisions about medical treatment is significantly impaired.

Indeed, mental illness is one of the main obstacle to medical decision making, and psychiatrists are usually involved in evaluating decisional capacity in hospitalized patients refusing medical therapies.

Therefore, compulsory treatment of COVID-19 deniers would of course count as a condition of exemption from the otherwise central jurisprudential principle of freedom of choice in the bioethical matter of medical treatment (15), whose driving concept (informed consent of the patient) is challenged by illness denial.

## Conclusions

Extreme societal reaction to COVID-19 pandemic included also denialism and conspiracy interpretations. Besides its mediatic, more or less instrumentally amplified impact^5^, such extreme reactions have clear public health effects at the societal level (e.g., reducing vaccination rate and delaying the reach of a possible herd immunity) as well as critical reverberations at individual level when infected patients refuse urgent lifesaving treatments for an illness they do not believe to exist.

While still not precisely quantified this phenomenon deserves an appropriate bioethical discussion which could be helpful not only along the current pandemic but also for possible future similar situations of societal and/or individual illness-denialism. In this perspective, bioethicists as well as psychiatrists must be aware of the challenge that the Anti-Vax movement is posing to the evaluation of extreme cultural beliefs, whose widespread diffusion may be enhanced by social media in current globalized and connected western society, especially when they harbor a clear potential for a huge impact in terms of public safety and individual decision-making (16).

Given the consequences that the involvement of a psychiatric assessment could have in the decision about involuntary treatment of hospitalized COVID-19 deniers in need of urgent, life-saving intervention, it is desirable to formalize such decision at political-administrative level after due ethical, medical and public health debate. Political authorities might decide about vaccine obligation while maintaining freedom of choice in end-of-life decisions.

## Author Contributions

All authors equally conceived the paper, analyzed the current situation and wrote the first draft, revised it and contributed to the final manuscript, and agreed on the final version of the manuscript.

## Conflict of Interest

The authors declare that the research was conducted in the absence of any commercial or financial relationships that could be construed as a potential conflict of interest.

## Publisher's Note

All claims expressed in this article are solely those of the authors and do not necessarily represent those of their affiliated organizations, or those of the publisher, the editors and the reviewers. Any product that may be evaluated in this article, or claim that may be made by its manufacturer, is not guaranteed or endorsed by the publisher.

## Footnotes

^1^Available online at: https://www.telegraph.co.uk/global-health/science-and-disease/vaccines-carry-tiny-knives-cut-veins-inside-romanias-toxic-anti/

^2^Available online at: https://www.kwch.com/2021/09/29/patients-refusal-treatment-creates-new-challenge-hospitals/

^3^Available online at: https://www.timesofisrael.com/1500-said-to-refuse-covid-antibody-treatment-leading-to-preventable-deaths/

^4^Available online at: https://www.siaarti.it/news/622309

^5^Available online at: https://www.wsj.com/articles/russian-disinformation-campaign-aims-to-undermine-confidence-in-pfizer-other-covid-19-vaccines-u-s-officials-say-11615129200
